# Phytochemical Profiling and Neuropharmacological Evaluation of *Baccaurea motleyana* Bark Extract: In Vivo and In Silico Approaches

**DOI:** 10.1155/tswj/9799401

**Published:** 2026-08-25

**Authors:** Md. Tanveer Ahsan, Md. Ekramul Haque Ekram, Kawsar Al Mahmud, Tanvir Hasan, Md. Mahmudul Hasan

**Affiliations:** ^1^ Department of Pharmacy, Faculty of Biological Sciences, University of Chittagong, Chittagong, Bangladesh, cu.ac.bd; ^2^ Department of Pharmacy, International Islamic University Chittagong, Chattogram, Bangladesh, iiuc.ac.bd; ^3^ Department of Pharmacy, East West University, Dhaka, Bangladesh, ewubd.edu

## Abstract

Depression, anxiety, insomnia, and muscle spasms are globally prevalent disorders. Although *Baccaurea motleyana* is traditionally used in folk medicine, its neuroprotective efficacy remains unexplored. This study evaluates the anxiolytic, antidepressant, sedative, and muscle relaxant properties of its bark methanol extract (BMB‐ME) using in vivo and in silico analyses. Phytochemical analysis of BMB‐ME was assessed by gas chromatography–mass spectrometry (GC‐MS). Antidepressant activity was evaluated using the tail suspension test (TST) and forced swim test (FST), anxiolytic effects were assessed using the elevated plus maze (EPM) and hole board test (HBT), sedative effects were measured using the open field test (OFT) and hole cross test (HCT), and muscle relaxant activity was determined using the rotarod test. Moreover, molecular docking and ADMET analyses were performed. GC‐MS of BMB‐ME identified 38 compounds. It significantly reduced immobility in TST to 61.75 ± 1.87 s (*p* < 0.001) and in FST to 55.52 ± 3.14 s (*p* < 0.001) at 400 mg/kg. EPM results showed increased open arm time to 232.6 ± 7.24 s (*p* < 0.001), whereas OFT demonstrated reduced locomotion to 45.6 ± 1.92 (*p* < 0.001). Rotarod retention time decreased to 18.6 ± 0.99 s (*p* < 0.001), indicating muscle relaxation. Molecular docking showed favorable binding affinities for hexadecanoic acid and gamma‐sitosterol (−8.3 to −8.1 kcal/mol), providing computational support. Overall, BMB‐ME demonstrated preliminary antidepressant‐, anxiolytic‐, sedative‐, and muscle relaxant–like effects in experimental models. Further studies are needed to isolate active compounds and clarify the underlying mechanisms.

## 1. Introduction

Depression and anxiety are widespread mental health disorders that significantly impact worldwide well‐being [1]. Depression is characterized by persistent sadness, reduced interest, and impaired daily functioning, affecting around 332 million people worldwide, with a greater prevalence among women [2]. Anxiety disorders are defined by disproportionate fear or tension that interrupts routine tasks, affecting approximately 359 million people globally, with a 55% growth in cases from 1990 to 2019 [3, 4]. Sedation, generally attained via pharmaceutical drugs, is utilized to calm patients or facilitate medical procedures; nonetheless, it carries risks such as dependence and respiratory depression [5]. COVID‐19 pandemic exacerbated these conditions, leading to a 25% increase in prevalence, particularly among adolescents and vulnerable populations, including tribal communities, who demonstrated notably elevated rates [6]. Depression and anxiety impose an annual economic burden of nearly US$1 trillion globally, emphasizing the need for improved mental health care. Muscle spasms are involuntary muscle contractions causing pain and stiffness and affect over 37% of healthy individuals annually [7]. The incidence of muscle spasms increases with age, affecting up to 50% of individuals aged ≥ 60 years and often disrupting sleep and reducing quality of life [8]. Conventional treatments for depression, anxiety, sedation, and muscle spasms include SSRIs, SNRIs, TCAs, benzodiazepines, sedative–hypnotics, and muscle relaxants [9]. Although effective, these agents are associated with limitations such as delayed therapeutic response, sexual dysfunction, weight gain, dependence, sedation, cognitive impairment, respiratory depression, and reduced long‐term tolerability. These challenges highlight the need for careful clinical use and continued exploration of alternative therapeutic approaches [10–13].

*Baccaurea motleyana* (Rambai) is a tropical fruit‐bearing plant widely distributed throughout Southeast Asia and is valued for both its nutritional and medicinal properties [14]. Phytochemical investigations have revealed the presence of diverse bioactive constituents, including phenolic compounds, flavonoids, terpenoids, and other antioxidant molecules that contribute to its significant free radical scavenging activity [15]. These compounds are of particular pharmacological interest because phenolics and flavonoids have been extensively reported to modulate central nervous system function through interactions with neurotransmitter pathways, regulation of neuroinflammation, and protection against oxidative damage [16]. Accumulating evidence suggests that oxidative stress plays a critical role in the pathogenesis of anxiety, depression, and other neurobehavioral disorders by disrupting neuronal signaling, impairing synaptic plasticity, and promoting neuronal injury [17]. Therefore, agents possessing strong antioxidant properties may alleviate these disorders by restoring redox balance and protecting neuronal integrity. Furthermore, molecular docking studies of *B. motleyana* have identified several phytoconstituents with potential affinity toward serotonergic and GABAergic targets, two major neurotransmitter systems involved in the regulation of mood, anxiety, sedation, and muscle relaxation [18]. The combined evidence of its bioactive composition, antioxidant capacity, and predicted interactions with neuropharmacological targets provides a strong scientific rationale for investigating the neurobehavioral effects of *B. motleyana*.

Computer‐aided drug design (CADD) supports drug discovery by using computational methods to identify potential therapeutic agents. Molecular docking predicts ligand–protein interactions and estimates binding affinity, enabling rapid and cost‐effective compound screening. CADD also integrates ADMET (absorption, distribution, metabolism, excretion, and toxicity) analysis to assess pharmacokinetic and toxicity profiles early in development, helping prioritize promising candidates and reduce late‐stage drug failure [19].

Despite the growing evidence about the antioxidant and neuromodulatory properties of *B. motleyana*, there are few comprehensive studies examining its effects on mood regulation, anxiety, and depression, employing both in vivo behavioral models and in silico mechanistic research. This research checks the neuromodulatory implications of *B. motleyana* bark extract by examining its anxiolytic, antidepressant, and sedative properties in animal models, as well as conducting molecular docking and ADMET analyses to identify and characterize active metabolites and their mechanisms of action, thus establishing a scientific foundation for its traditional application in addressing stress‐related neurological disorders.

## 2. Materials and Methods

### 2.1. Chemicals and Reagents

All chemicals and reagents used in this study were of analytical grade and were supplied by the Department of Pharmacy, University of Chittagong. Tween 80, methanol (99.9% purity), fluoxetine, and diazepam were employed in the experimental procedures. Diazepam and fluoxetine were procured from Square Pharmaceuticals PLC. Methanol and Tween 80 were obtained from Sigma‐Aldrich, St. Louis, Missouri, United States. The use of analytical‐grade reagents ensured the precision, consistency, and reproducibility of the experimental outcomes.

### 2.2. Collection and Extraction

A total of 1.5 kg of *B. motleyana* bark was procured from Narsingdi, Bangladesh, in August 2024. Professor Dr. Shaikh Bokhtear Uddin of the Department of Botany at the University of Chittagong authenticated and cataloged the plant sample as MMH‐050824. After collection, the bark was purified, sectioned, air‐dried at ambient temperature for 10 days, and then oven‐dried for 24 h to facilitate grinding. A high‐capacity grinder pulverized dried bark into coarse powder. In a neat round‐bottom flask, 650 g of powdered bark was infused in 4 L of methanol, covered with aluminum foil, and extracted for 14 days with intermittent agitation. A cotton outlet, Whatman No. 1 filter paper, and a vacuum filtration accessory were used to filter the mixture gradually. A Buchi rotary evaporator, operating at lower pressure and below 50°C, concentrated the filtrate to yield 12 g of crude *B. motleyana* bark methanol extract (BMB‐ME) with an extraction efficiency of 1.85%, which was stored at 4°C until required [20].

### 2.3. Preliminary Phytochemical Screening

Qualitative phytochemical study of BMB‐ME identified bioactive flavonoids, phenolics, alkaloids, and terpenoids using conventional methodologies described by Adusei et al. [21].

### 2.4. GC‐MS Analysis

Following the procedure described by Naveed et al., BMB‐ME was analyzed using a Shimadzu GC‐17A gas chromatograph coupled with a TQ‐8040 mass spectrometer operating in electron ionization mode. Helium was used as the carrier gas at a constant flow rate of 0.6 mL/min. The interface temperature was maintained at 280°C, and the mass spectra were recorded over a scan range of 40–350 amu. Identification of the detected compounds was carried out by comparing the obtained spectra with those in the NIST GC MS Library Version 08 S [22].

### 2.5. Animal Experiments and Ethics

Female Swiss albino mice, weighing 25–35 g and aged 4–5 weeks, were utilized in this experiment. Five mice were accommodated in typical plastic containers with steel‐grid lids under controlled conditions: a 12‐h light–dark cycle, 25^°^C ± 2^°^C, and 45%–55% relative humidity. The sample size (*n* = 5 per group) was selected based on commonly accepted practices in preliminary in vivo neuropharmacological screening studies and in accordance with the principle of reduction under the 3Rs (replacement, reduction, and refinement) to minimize animal use while maintaining sufficient statistical power to detect treatment‐related differences [23]. Food and water were provided ad libitum throughout the experimental period. The Animal Ethics Review Board of the University of Chittagong, Faculty of Biological Sciences, sanctioned all experiments (AERB‐FBSCU‐20250615‐(1)).

### 2.6. Acute Toxicity

The acute ingestion toxicity of BMB‐ME was assessed according to OECD 425 guidelines. A 2000 mg/kg oral dose was administered to a mouse following a 3‐h fast. The mouse was observed for signs of poisoning over 24 h, including appearance, behavior, respiration, and central nervous system function. Four additional mice were subjected to treatment and observed for a duration of 14 days [24].

### 2.7. CNS Antidepressants

#### 2.7.1. Tail Suspension Test (TST)

The TST was conducted according to the procedures described by Cryan. Mice were randomly assigned to four groups: a control group, a standard group treated with fluoxetine at 2.5 mg/kg, and two test groups receiving BMB‐ME at 200 and 400 mg/kg. All treatments were administered at 24, 18, and 1 h before the experiment. During the test, each mouse was suspended by the tail, and the duration of immobility was recorded over the observation period. A reduction in immobility time compared with the control group was considered indicative of an antidepressant‐like effect [25].

#### 2.7.2. Forced Swimming Test (FST)

According to the method described by Mohammad et al., the FST was conducted to evaluate the antidepressant potential of BMB‐ME. Four groups of five mice each were included in the study. The animals received either BMB‐ME at doses of 200 and 400 mg/kg, a standard antidepressant drug (fluoxetine 2.5 mg/kg), or control treatment. Each mouse was placed individually in a water‐filled cylinder, and the duration of immobility was recorded over the observation period. The time spent immobile served as an indicator of behavioral despair. A reduction in immobility time compared with the control group was considered to reflect antidepressant‐like activity and was used to determine the potency of the extract [26].

### 2.8. Anxiolytic Profiling

#### 2.8.1. Elevated Plus‐Maze (EPM) Test

The experimental procedures followed the guidelines described by Violle et al. A total of four groups were included in the study, with five mice randomly allocated to each group. The animals were assigned to control, standard, and test groups. The test groups received BMB‐ME at 200 and 400 mg/kg, the standard group received diazepam (2 mg/kg), and the control group received the vehicle. Behavioral assessments were conducted using the TST, the FST, and the EPM test. In the EPM, each mouse was placed at the center of the apparatus facing an open arm, and the number of entries and time spent in open and closed arms were recorded [27].

#### 2.8.2. Hole Board Test (HBT)

The HBT was conducted following the procedure described by Violle et al. Four groups of five mice each were randomly assigned to control, standard, or test groups. The test groups received BMB‐ME at 200 and 400 mg/kg, the standard group received diazepam orally at 2 mg/kg, and the control group received the vehicle. During the test, each mouse was placed individually on the hole board, and the number of head‐dipping behaviors into the holes was recorded over the observation period [27].

### 2.9. Sedative Profile

#### 2.9.1. Hole Cross Test (HCT)

The HCT was performed following the guidelines described by Afrin et al., using the specified apparatus [28]. Five mice were randomly assigned to four groups: BMB‐ME at 200 or 400 mg/kg, the standard drug diazepam (2 mg/kg), and the control vehicle. During the test, the number of crossings between compartments was recorded. The percentage of locomotor activity inhibition was calculated using the following formula:

Movement inhibition %=no.of movement control−no.of movement testno.of movement control×100.

#### 2.9.2. Open Field Test (OFT)

The OFT was conducted according to the methodology described by Eva et al. [29]. Four groups of mice were randomly assigned to receive either the control vehicle, the standard drug diazepam (2 mg/kg), or BMB‐ME at doses of 200 or 400 mg/kg. Each mouse was placed individually in the open field apparatus, and the number of squares crossed was recorded as a measure of locomotor activity. Observations were made before treatment and at 30, 60, 90, and 120 min after treatment.

### 2.10. Muscle Relaxation

#### 2.10.1. Rotarod Test

The rotarod test was conducted according to the methodology described by Afrin et al. [28]. Four groups of mice (*n* = 5) were randomly assigned to receive either the control vehicle, the standard drug diazepam (2 mg/kg), or BMB‐ME at doses of 200 and 400 mg/kg. After treatment, each mouse was placed on the rotating rod, and the fall‐off time was recorded at 30, 60, 90, and 120 min. The percentage reduction in fall‐off time was calculated using the formula:

Reduction in fall−off time %=A−BA×100.

Here, *A* refers to the fall‐off time pretreatment, and *B* refers to the fall‐off time posttreatment.

### 2.11. In Silico Studies

#### 2.11.1. Protein Preparation

Three target proteins were selected for molecular docking studies: the human serotonin transporter (PDB ID: 5I6X) to evaluate antidepressant activity, the human GABAA receptor (PDB ID: 6X3W) for anxiolytic and sedative effects, and the human M1 muscarinic acetylcholine receptor (PDB ID: 5CXV) for muscle relaxation [30]. The three‐dimensional structures of these proteins were obtained from the RCSB Protein Data Bank. Water molecules and heteroatoms were removed using BIOVIA Discovery Studio Visualizer, and energy minimization was performed with Swiss‐PdbViewer to optimize the structures for docking analysis.

#### 2.11.2. Ligand Preparation

Metabolites identified in the GC‐MS analysis of BMB‐ME were retrieved in 3D SDF format from PubChem. For compounds lacking 3D structures, Open Babel was used to generate 3D conformations from 2D structures. All ligands were then prepared in PyRx by converting them to PDBQT format and performing energy minimization using the MMFF94 force field to ensure stable conformations for molecular docking studies [31].

#### 2.11.3. Molecular Docking

Molecular docking was performed in PyRx using AutoDock Vina. Proteins were kept rigid, whereas ligands were allowed up to 10° of conformational flexibility. Docking protocols were validated by redocking the cocrystallized ligands within grid boxes centered on the active sites, with RMSD values below 2 Å considered acceptable. The final docking grid coordinates and validation results were as follows: serotonin transporter (5I6X)—*X* = 40.87, *Y* = 24.95, and *Z* = −15.01 (RMSD = 0.78 Å); GABAA receptor (6X3W)—*X* = 131.08, *Y* = 113.54, and *Z* = 144.12 (RMSD = 1.02 Å); and M1 muscarinic receptor (5CXV)—*X* = −23.92, *Y* = −48.73, and *Z* = 185.62 (RMSD = 0.54 Å). The docking interactions, both two‐dimensional and three‐dimensional, were visualized using BIOVIA Discovery Studio Visualizer 2020 [32].

#### 2.11.4. Drug‐Likeness and ADMET Analyses

Pharmacokinetic properties, including ADMET, as well as drug likeness, were evaluated using pkCSM and SwissADME. Lipinski′s rule of five was applied to identify compounds with favorable medicinal and pharmacological potential [33].

### 2.12. Statistical Analysis

All results are presented as mean ± SEM. Statistical analyses were performed using SPSS (Version 25) and GraphPad Prism (Version 5.0). Data are expressed as mean ± SEM. For single‐time point experiments (TST, FST, EPM, and HBT), group differences were analyzed using one‐way ANOVA followed by Dunnett′s multiple comparison test versus control. Time‐course data (OFT, HCT, and rotarod) were analyzed using a two‐way repeated‐measures ANOVA with factors of treatment and time, followed by a Bonferroni post hoc correction. Normality and homogeneity of variances were assessed using the Shapiro–Wilk and Levene tests, respectively. Statistical significance was set at *p* < 0.05, and exact *p* values are reported in tables and figures [34].

## 3. Results

### 3.1. Phytochemical Screening

#### 3.1.1. Preliminary Phytochemical Screening

Preliminary phytochemical screening of BMB‐ME confirmed the presence of several bioactive compounds. Flavonoids, alkaloids, saponins, phenols, and tannins were detected, with saponins showing a strong reaction. Phytosterols, glycosides, cardiac glycosides, and anthraquinones were also present. Steroids were found in low amounts, whereas terpenoids were absent. Resins, proteins, and some carbohydrates showed positive results in selected tests (Table 1).

**Table 1:** Qualitative phytochemical screening of BMB‐ME.

Phytochemicals	Methods	BMB‐ME
Flavonoids	Alkaline reagent test	+
	Zinc–hydrochloric acid reduction test (flavanol)	++
	Lead acetate solution test	+
	Sulfuric acid test	++

Alkaloids	Mayer′s test	++
	Wagner′s test	++
	Hager′s test	++

Saponins	Foam test	+++

Phenols and tannins	Ferric chloride test	++

Steroids	Salkowski′s test	+
	Liebermann–Burchard′s test	−

Terpenoids	Salkowski′s test	−
	Liebermann–Burchard′s test	−

Phytosterols	Liebermann–Burchard′s test	++

Glycosides	Liebermann′s test	++
	Salkowski′s test	++
	Sodium hydroxide reagent test	−

Cardiac glycosides	Keller–Killiani′s test	+
	Baljet′s test	+

Anthraquinones	Acid test	++
	Hydroxyanthraquinone test	++

Phenolic derivatives (phlobatannins)	Hydrochloric acid test	+

Resins	Acetone test	++

Proteins	Biuret test	+
	Nitric acid test	++

Carbohydrates	Molisch′s test	+
	Fehling′s test	+
	Benedict′s test	−
	Iodine test	−

Fats and fixed oils	Copper sulfate test	−

Coumarins	Sodium hydroxide test	−

*Note:* “+” = present; “++” = moderately present; “+++” = abundantly present; “−” = absent.

Abbreviation: BMB‐ME, *B. motleyana* bark methanolic extract.

#### 3.1.2. GC‐MS Analysis of BMB‐ME

**Figure 1:**
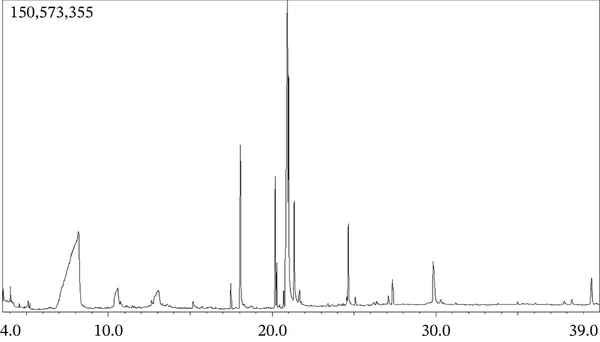
GC‐MS chromatogram of the BMB‐ME. GC‐MS analysis of the BMB‐ME revealed 38 metabolites, detected between 3.70 and 39.5 min.

GC‐MS analysis of BMB‐ME revealed the presence of several biologically active compounds. The characteristic chromatogram is shown in Figure 1. The identified constituents are listed in Table 2, along with their retention times, molecular weights, and peak area percentages.

**Table 2:** GC‐MS‐identified major bioactive compounds of BMB‐ME with their retention times, molecular weights, and relative concentrations.

Serial no.	Compound name	Retention time (min)	Concentration (%)
1	E,Z‐1,3,12‐nonadecatriene	20.931	31.64
2	Glycerin	8.150	24.73
3	*n*‐Hexadecanoic acid	18.054	5.50
4	L‐arabinitol	13.097	3.46
5	Erythritol	10.554	2.92
6	9,12‐octadecadienoic acid (Z, Z)‐, 2,3‐dihydroxypropyl ester	29.840	2.09
7	Stigmast‐4‐en‐3‐one	22.545	1.43
8	Gamma‐sitosterol	39.484	1.08
9	Asperglaucide	25.500	0.93
10	(E)‐4‐(3‐hydroxyprop‐1‐en‐1‐yl)‐2‐methoxyphenol	15.165	0.75

### 3.2. Acute Toxicity Investigation

Acute oral toxicity of BMB‐ME was evaluated in accordance with OECD guideline 425. Both male and female mice were used in the study. A single fasted mouse was initially administered the extract orally at a dose of 2000 mg/kg and observed for 24 h for any signs of toxicity, including changes in skin, fur, eyes, mucous membranes, circulation, respiration, autonomic activity, and central nervous system function. After no adverse effects or mortality were observed, four additional fasted mice received the same dose and were monitored for 14 days for behavioral changes, signs of toxicity, or death. No mortality or significant adverse effects were observed in any of the animals, indicating that the LD_50_ of BMB‐ME exceeds 2000 mg/kg. Based on this, the therapeutic doses for pharmacological testing were set at one‐tenth (200 mg/kg) and one‐fifth (400 mg/kg) of the limit dose [35].

### 3.3. Evaluation of Antidepressant Activity

#### 3.3.1. TST and FST

**Figure 2:**
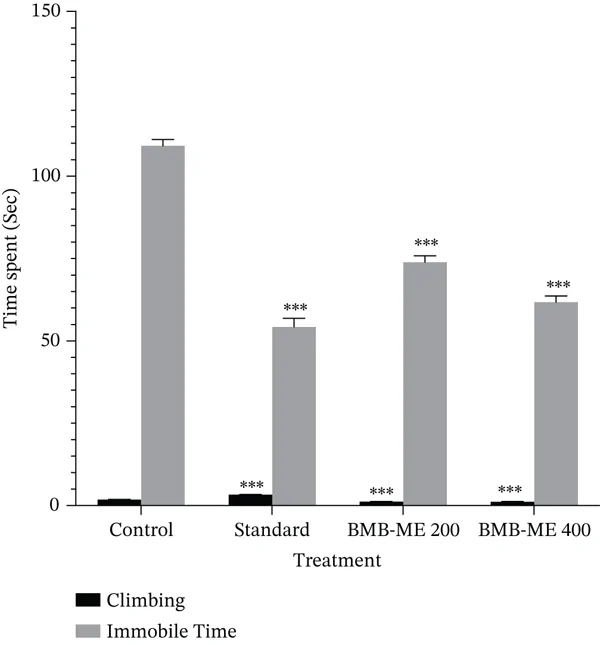
Effect of MBM‐ME on the immobility time in TST. Compared with the control group, statistically significant differences were indicated by  ^∗∗∗^ *p* < 0.001. Values are presented as mean ± SEM (*n* = 5).

**Figure 3:**
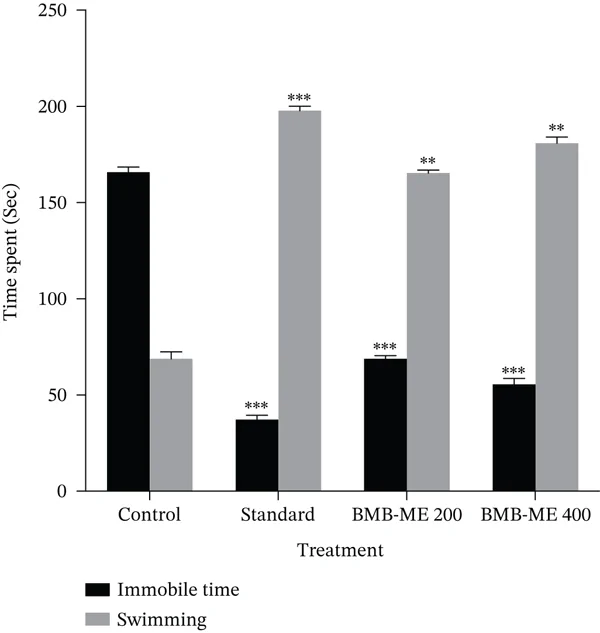
Impact of BMB‐ME on the immobility time in FST. Compared with the control group, statistically significant differences were indicated by  ^∗∗^ *p* < 0.01 and  ^∗∗∗^ *p* < 0.001. The values were presented as mean ± SEM (*n* = 5).

The antidepressant‐like activity of BMB‐ME was assessed by implementing the TST and the FST. During TST, control mice had an immobility duration of 109.18 ± 1.97 s, which was considerably diminished by the usual medication to 54.2 ± 2.63 s (*p* < 0.001). BMB‐ME at 200 and 400 mg/kg decreased immobility to 73.9 ± 1.91 and 61.75 ± 1.87 s, respectively, with a corresponding significant rise in climbing behavior (*p* < 0.001) (Figure 2). In FST, control animals exhibited immobility of 165.81 ± 2.63 s and swimming of 68.73 ± 3.66 s. Treatment with fluoxetine reduced immobility to 37.25 ± 2.26 s and increased swimming time to 197.7 ± 2.35 s. BMB‐ME at 200 and 400 mg/kg significantly decreased immobility to 68.82 ± 1.64 and 55.52 ± 3.14 s while enhancing swimming to 165.29 ± 1.62 and 180.84 ± 3.18 s, respectively (*p* < 0.001) (Figure 3).

### 3.4. Evaluation of Sedative Activity

#### 3.4.1. OFT and HCT

**Figure 4:**
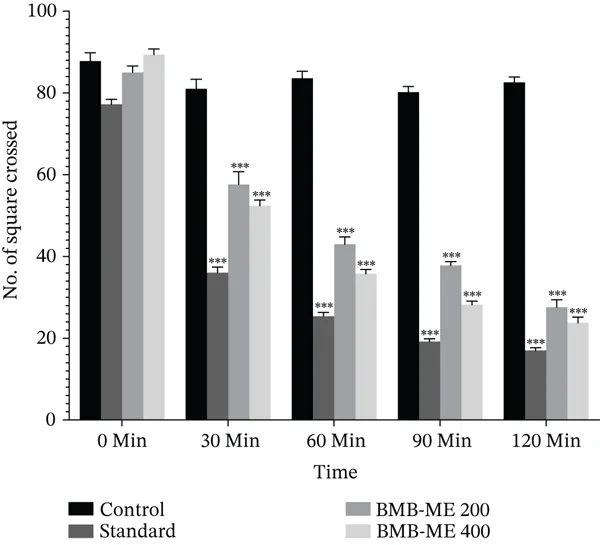
Impact of BMB‐ME on murine subjects in OFT. Compared with the control group, statistically significant differences were indicated by  ^∗∗∗^ *p* < 0.001. Values are presented as mean ± SEM (*n* = 5).

**Figure 5:**
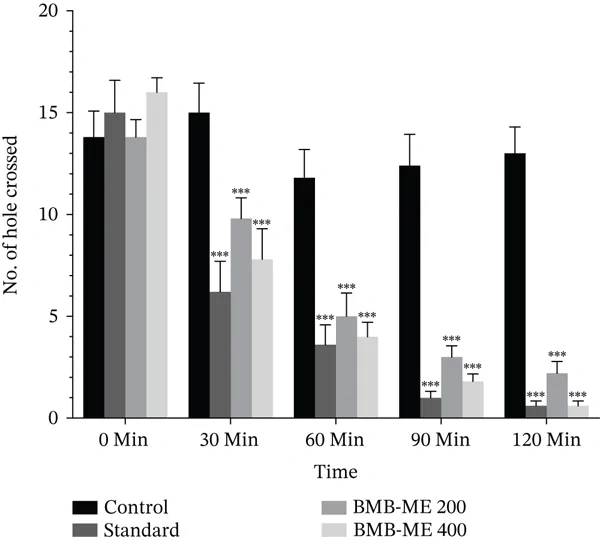
Effect of BMB‐ME on mice in HCT. Compared with the control group, statistically significant differences were indicated by  ^∗∗∗^ *p* < 0.001. Values were presented as mean ± SEM (*n* = 5).

The sedative and CNS depressant effects of BMB‐ME were evaluated using the OFT and HCT. In the OFT, control mice exhibited stable locomotor activity throughout the observation period, whereas diazepam significantly reduced activity (*p* < 0.001). BMB‐ME at 200 mg/kg moderately decreased locomotion, while 400 mg/kg generated a more noticeable reduction, approaching the impact of diazepam (*p* < 0.001). Similarly, the duration of immobility increased significantly with BMB‐ME treatment in a dose‐dependent manner (*p* < 0.001) (Figure 4). In the HCT, control animals maintained normal exploratory behavior, whereas diazepam markedly reduced the number of hole crosses (*p* < 0.001). BMB‐ME at 200 mg/kg caused a moderate decrease in exploratory activity. In contrast, 400 mg/kg significantly inhibited movement, comparable to diazepam (*p* < 0.001) (Figure 5).

### 3.5. Evaluation of Anxiolytic Activity

#### 3.5.1. EPM Test

**Figure 6:**
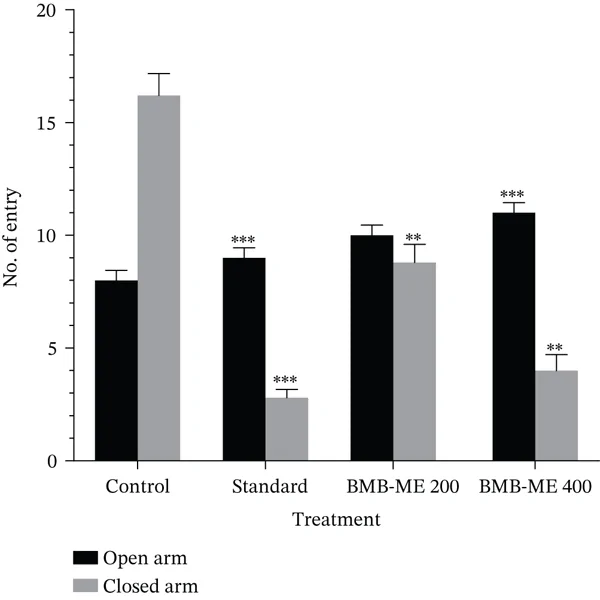
Impact of BMB‐ME on the number of entries in EPM. Compared with the control group, statistically significant differences were indicated by  ^∗∗^ *p* < 0.01 and  ^∗∗∗^ *p* < 0.001. Values are presented as mean ± SEM (*n* = 5).

**Figure 7:**
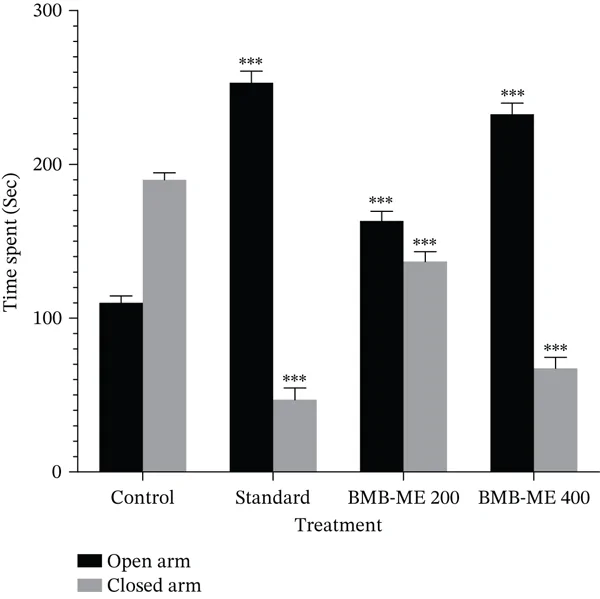
Impact of BMB‐ME on duration of time allocated in EPM. Compared with the control group, statistically significant differences were indicated by  ^∗∗∗^ *p* < 0.001. Values are presented as mean ± SEM (*n* = 5).

BMB‐ME at 200 and 400 mg/kg significantly increased both the frequency of entry and the duration spent in the open arms of the EPM relative to control subjects (*p* < 0.0001). Specifically, BMB‐ME at 200 mg/kg produced 10 ± 0.45 entries and 163.2 ± 6.38 s spent in the open arms, whereas the 400 mg/kg dose resulted in 11 ± 0.45 entries and 232.6 ± 7.24 s, approaching the effect of diazepam (9 ± 0.45 entries and 253 ± 7.64 s) (Figures 6 and 7).

#### 3.5.2. HBT

**Figure 8:**
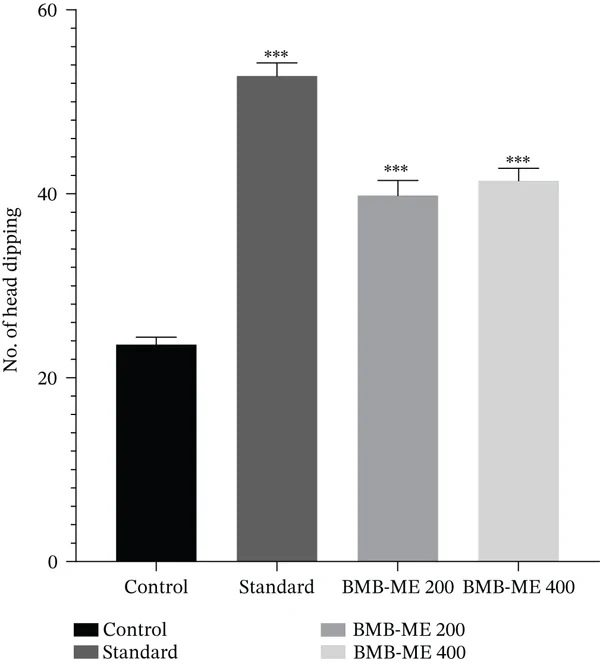
Effect of BMB‐ME on the number of head dips in HBT. Compared with the control group, statistically significant differences were indicated by  ^∗∗∗^ *p* < 0.001. The values were presented as mean ± SEM (*n* = 5).

During the HBT, BMB‐ME markedly elevated the frequency of head dips relative to the control (23.6 ± 0.81). Treatment with 200 and 400 mg/kg resulted in head dip counts of 39.8 ± 1.66 and 41.4 ± 1.36, respectively, whereas diazepam increased the count to 52.8 ± 1.43 (*p* < 0.0001). These findings indicate that BMB‐ME exerts a dose‐dependent anxiolytic effect comparable to fluoxetine (Figure 8).

### 3.6. Evaluation of Muscle Relaxant Activity

#### 3.6.1. Motor‐Coordination Test (Rotarod Test)

**Figure 9:**
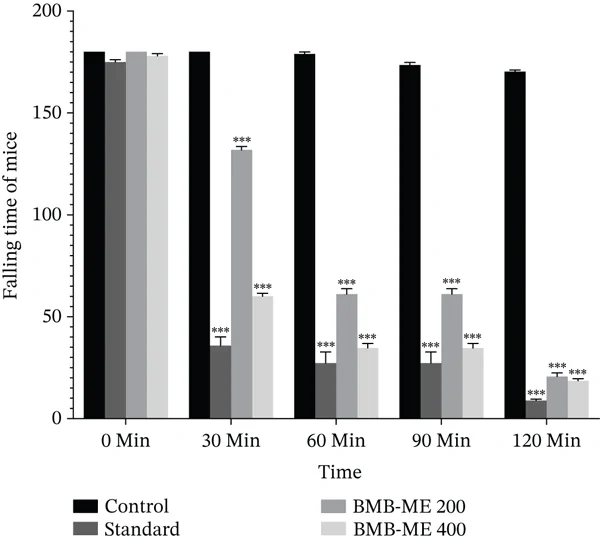
Assessment of the muscle relaxant efficacy of BMB‐ME. Compared with the control group, statistically significant differences were indicated by  ^∗∗∗^ *p* < 0.001. The values were presented as mean ± SEM (*n* = 5).

The muscle relaxant effect of BMB‐ME was evaluated using the rotarod test. Control mice maintained relatively stable fall times throughout the 120‐min observation period, indicating regular motor coordination. Diazepam significantly reduced fall‐off time at all time points, from 175 ± 1.12 s at 0 min to 8.85 ± 0.66 s at 120 min (*p* < 0.001), indicating sedation and impaired motor coordination. BMB‐ME at 200 mg/kg progressively decreased fall‐off time from 180 ± 0 to 20.63 ± 1.84 s, whereas the 400 mg/kg dose caused a greater reduction from 178 ± 1.12 to 18.6 ± 0.99 s over the same period (*p* < 0.001), consistent with dose‐dependent muscle relaxation and motor impairment (Figure 9).

### 3.7. Molecular Docking Study

**Figure 10:**
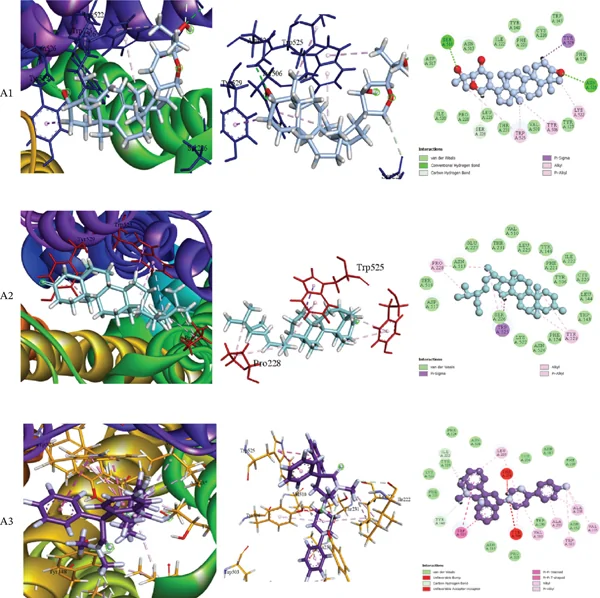
Illustrations of the most prominent docked molecules and standard drug with the human serotonin transporter (PDB ID: 5I6X). (A) Human serotonin transporter–hexadecanoic acid complex, (B) human serotonin transporter–gamma‐sitosterol complex, and (C) human serotonin transporter–fluoxetine (standard) complex.

**Figure 11:**
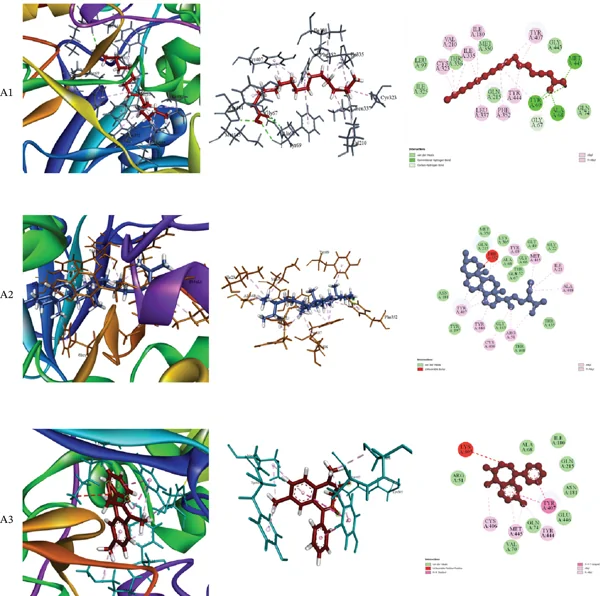
Illustrations of the most prominent docked molecules using the human GABAA receptor (PDB ID: 6X3W). (A) Hexadecenoic acid–human GABAA receptor complex, (B) gamma‐sitosterol–human GABAA receptor complex, and (C) diazepam (standard)–human GABAA receptor complex.

**Figure 12:**
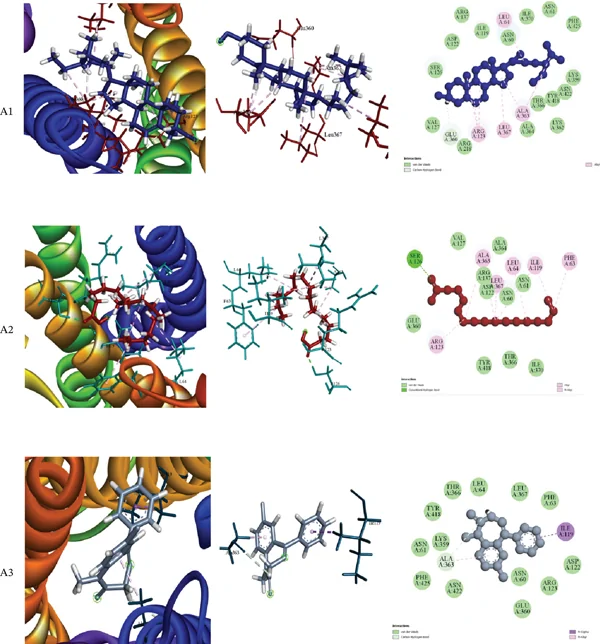
Illustrations of the top docked compounds and standard with human M1 muscarinic acetylcholine receptor (PDB ID: 5CXV). (A) M1 muscarinic acetylcholine receptor–gamma‐sitosterol complex, (B) M1 muscarinic acetylcholine receptor–hexadecanoic acid complex, and (C) diazepam–M1 muscarinic acetylcholine receptor complex.

Table 3 outlines the molecular docking assessment of BMB‐ME phytoconstituents on three key pharmacological receptors of focus. Figures 10, 11, and 12 illustrate the top‐ranking molecule for each protein.

**Table 3:** Binding affinity between the lead chemicals and standards against target proteins.

Receptor	Compound	Binding affinity (kcal/mol)	Bond type	Amino acids
5I6X	Hexadecanoic acid	−8.2	Alkyl	VAL501, ILE172(3)
			Pi–alkyl	TYR95, PHE335, PHE341(3)
	Gamma‐sitosterol	−8.1	Conventional hydrogen bond	ARG104
			Pi–alkyl	TYR95, TYR176(2), PHE335, PHE341
			Alkyl	ALA169, ALA173, ILE172(3)
	Fluoxetine	−8.2	Conventional hydrogen bond	TYR95
			Carbon hydrogen bond	ALA173, ASP98
			Halogen (fluorine)	ILE172, SER439
			Pi–pi T‐shaped	TYR176
			Amide–pi stacked	SER438, SER439
			Alkyl	ILE172
			Pi–alkyl	TYR176, ILE172(2), VAL501

6X3W	Hexadecanoic acid	−8.1	Conventional hydrogen bond	ALA68, TYR69(2), MET445
			Carbon hydrogen bond	GLY67
			Pi–alkyl	TYR69, PHE352, TYR407(2), TYR444
			Alkyl	ILE180, ILE335(2), LEU337, VAL210, CYS323
	Gamma‐sitosterol	−7.9	Alkyl	ALA448, ARG51(2), CYS406, ILE23, MET445
			Pi–alkyl	TYR69, PHE352(2), TYR407(3), TYR444
	Diazepam	−8.3	Alkyl	MET445, CYS406
			Pi–alkyl	TYR444, TYR407, MET445
			Pi–pi T‐shaped	TYR407
			Pi–pi stacked	TYR407

5CXV	Gamma‐sitosterol	−8.3	Alkyl	ARG123(3), ALA363(3), LEU367, LEU64
			Carbon hydrogen bond	GLU360
	Hexadecanoic acid	−8.1	Alkyl	ARG123, ALA363(2), ILE119(2), LEU367, LEU64
			Conventional hydrogen bond	SER126
			Pi–alkyl	PHE63
	Diazepam	−8.2	Carbon hydrogen bond	ALA363
			Pi–sigma	ILE119
			Pi–alkyl	ALA363

#### 3.7.1. Docking Study for Antidepressant Activity

Nonbond relationships between docking metrics and the drugs that correlate with antidepressant effectiveness are shown in Table 3. Our molecular docking inquiry involved docking each chemical to the intended proteins. Hexadecanoic acid (−8.2 kcal/mol), gamma‐sitosterol (−8.1 kcal/mol), and fluoxetine (standard) (8.2 kcal/mol) have the most prevalent binding power to the human serotonin transporter (PDB ID: 5I6X). Hexadecanoic acid (−8.2 kcal/mol) exhibited a binding strength comparable to that of fluoxetine, suggesting the potential effectiveness of antidepressants.

#### 3.7.2. Docking Study for Anxiolytic and Sedative Activity

The nonbond interactions and docking numbers for the sedative and anxiolytic effects of the tested medications with the human GABAA receptor (PDB ID: 6X3W) in the amino acid region are shown in Table 3. In our molecular docking assessment, the intended proteins were docked to each molecule. Hexadecenoic acid (−8.1 kcal/mol), gamma‐sitosterol (−7.9 kcal/mol), and diazepam (standard) (−8.2 kcal/mol) expressed the most possible binding affinities against the human GABAA receptor (PDB ID: 6X3W).

#### 3.7.3. Docking Study for Muscle Relaxant Activity

The docking indices and nonbond correlations of the compounds linked with their corresponding muscle relaxant actions are shown in Table 3. Every molecule was docked to specific proteins in our molecular docking analysis. The compounds demonstrating the most significant binding affinities are gamma‐sitosterol (−8.3 kcal/mol), hexadecanoic acid (−8.1 kcal/mol), and diazepam (standard) (−8.2 kcal/mol) for the human M1 muscarinic acetylcholine receptor (PDB ID: 5CXV).

### 3.8. ADMET and Drug‐Likeness Analyses

To assess their therapeutic potential, the core plant compounds of BMB‐ME were evaluated for pharmacokinetic properties and drug likeness. Table 3 summarizes that each of the compounds complied with Lipinski′s five rules, demonstrating favorable drug‐like properties, and showed no mutagenic or carcinogenic effects, including negative AMES results, thereby supporting their safety profile for further development (Table 4).

**Table 4:** ADMET analysis of the top interacted metabolites of BMB‐ME.

Compound name	Water solubility (log mol/L)	Intestinal absorption (% absorbed)	VDss (human) (log L/kg)	BBB permeability (log BB)	CYP3A4 substrate	Total clearance (log mL/min/kg)	AMES toxicity	Hepatotoxicity	Drug likeliness
*n*‐Hexadecanoic acid	−5.562	92.004	−0.543	−0.111	Yes	1.763	No	No	Yes
Gamma‐sitosterol	−6.773	94.464	0.193	0.781	Yes	0.628	No	No	Yes
Octadecanoic acid	−5.973	91.317	−0.528	−0.195	Yes	1.832	No	No	Yes
Gamma‐tocopherol	−7.602	90.043	0.732	0.739	Yes	0.821	No	No	Yes
*Cis*‐sinapyl alcohol	−1.838	92.941	0.161	−0.224	No	0.241	No	No	Yes
Stigmast‐4‐en‐3‐one	−6.212	98.556	−0.141	0.847	Yes	0.560	No	No	Yes
Ergosta‐5, 24 (28)‐dien‐3‐ol	−7.041	94.691	0.426	0.777	Yes	0.604	No	No	Yes
5,9,13,17‐tetramethyl‐4,8,12,16‐octadecatetraenoic acid	−6.105	91.787	−0.580	−0.036	Yes	1.827	No	Yes	Yes
9,12‐octadecadienoic acid (Z, Z)‐, 2,3‐dihydroxypropyl ester	−5.888	91.247	−0.334	−0.857	Yes	2.136	No	No	Yes
Campesterol	−6.818	94.757	0.290	0.771	Yes	0.572	No	No	Yes

## 4. Discussion

Ethnomedicinal herbs are routinely employed to address neurobehavioral problems. They are regarded as more secure than traditional anxiolytics and antidepressants, which frequently induce adverse effects and tolerance [36]. The neuropharmacological effects of BMB‐ME were investigated using behavioral assessments, molecular docking, and phytochemical analysis. The existence of saponins, flavonoids, alkaloids, tannins, steroids, phenols, glycosides, and anthraquinones was verified through phytochemical analysis [37]. GC‐MS analysis revealed 38 bioactive compounds, with E,Z‐1,3,12‐nonadecatriene, gamma‐sitosterol, *n*‐hexadecanoic acid, and hexadecenoic acid as the predominant components. These chemicals are documented to have neuroprotective, anti‐inflammatory, and antioxidant characteristics [38]. Acute toxicity studies established an acceptable LD_50_ of 2000 mg/kg, validating the chosen dosages of 200 and 400 mg/kg for therapeutic assessment [39].

TST and FST are established behavioral models used to assess antidepressant‐like activity by measuring behavioral despair, where reduced immobility reflects improved coping and enhanced monoaminergic neurotransmission, particularly in the serotonergic system [40]. BMB‐ME contains bioactive phytochemicals, including flavonoids, alkaloids, and sterols, which are known to modulate neurotransmitter systems and contribute to neurobehavioral effects. In this study, BMB‐ME significantly decreased immobility and increased active behaviors in both TST and FST, suggesting notable antidepressant‐like effects. Supporting these behavioral findings, in silico docking indicated that *n*‐hexadecanoic acid and gamma‐sitosterol, two major constituents of the extract, may interact with the human serotonin transporter (PDB ID: 5I6X) with binding energies of −8.2 and −8.1 kcal/mol, respectively. Although these results do not confirm a direct mechanism, they provide a preliminary indication of potential serotonergic modulation that could complement the observed behavioral effects [41, 42]. Furthermore, the antioxidant and neuroprotective properties of these substances may mitigate depression symptoms by diminishing oxidative and inflammatory stress in neural cells [43].

The extract′s sedative action was validated by the OFT and HCT, which evaluate central nervous system depressive effects by measuring decreases in locomotor and exploratory behavior. A dose‐dependent reduction in movement was noted, signifying central nervous system depression and amplification of inhibitory neurotransmission [44]. Molecular interactions corroborate this behavioral effect: Hexadecenoic acid and gamma‐sitosterol, principal components of the extract, exhibited significant binding affinity for the human GABAA receptor (PDB ID: 6X3W), with docking scores of −8.1 and −7.9 kcal/mol, respectively. These interactions may enhance GABAergic signaling, resulting in neuronal hyperpolarization and reduced neuronal activity. Hexadecenoic acid, known for its effects on membrane integrity and ion channel function, may also enhance the calming effect by promoting inhibitory neurotransmission [45]. Because BMB‐ME also reduced locomotor activity in the OFT and HCT, the influence of sedation on behavioral outcomes should be considered. Reduced locomotion may affect performance in antidepressant and anxiolytic tests. However, the observed decrease in immobility in TST/FST and increased exploratory behavior in EPM/HBT suggest that the effects were not solely due to locomotor suppression, although further studies are needed to confirm this distinction [46].

The anxiolytic attributes of the extract were evidenced by EPM and HBT, which are recognized models for evaluating anxiety‐related behavior predicated on an animal′s inherent aversion to open environments. In time spent in open arms and a greater frequency of head dips, less anxiety and improved exploratory activity are observed [47]. This behavioral reaction aligns with the modulatory actions of the GABAA receptor, in which significant substances, including hexadecenoic acid and gamma‐sitosterol, exhibit strong binding, enhancing inhibitory neurotransmission and diminishing neuronal excitability [48]. Furthermore, E,Z‐1,3,12‐nonadecatriene, the predominant molecule in the extract, may enhance the anxiolytic effect through its antioxidant activity and membrane‐stabilizing properties, thereby promoting neuronal protection and tranquilizing responses [49].

The extract′s muscle relaxant effect was evaluated using the rotarod test, which measures neuromuscular coordination and motor performance. A dose‐dependent reduction in retention time was observed, indicating decreased motor coordination consistent with central muscle relaxant and sedative effects. In silico docking suggested potential interactions of gamma‐sitosterol and *n*‐hexadecanoic acid with the human M1 muscarinic acetylcholine receptor, with binding energies of −8.3 and −8.1 kcal/mol, respectively. Although these results indicate possible receptor binding, the exact mechanism by which BMB‐ME affects motor coordination remains speculative. The known membrane‐stabilizing and anti‐inflammatory properties of gamma‐sitosterol, along with the potential effects of *n*‐hexadecanoic acid on neuronal membrane dynamics, may contribute to the observed muscle relaxant activity [50–53].

ADMET analysis revealed advantageous pharmacokinetic characteristics of the principal compounds, including elevated gastrointestinal absorption (> 90%), sufficient water solubility, permeability across the blood–brain barrier, and complete adherence to Lipinski′s rule, signifying excellent oral bioavailability and central nervous system penetration [54, 55]. The lack of anticipated mutagenic or hepatotoxic effects further substantiates their safety for therapeutic use. The pharmacokinetic characteristics, along with robust receptor‐binding affinities, support the conclusion that the extract has neuromodulatory effects by interacting with essential neurotransmitter systems, including serotonergic (5I6X), GABAergic (6X3W), and cholinergic (5CXV) pathways. This multimodal approach efficiently addresses the neurochemical imbalances associated with sadness, anxiety, drowsiness, and muscle spasms. The findings substantiate the conventional use of *B. motleyana* as a neurotherapeutic agent and underscore its potential to advance to a therapeutically significant botanical preparation. Additional isolation of active chemicals and prolonged safety assessments are necessary to determine its medicinal potential.

## 5. Conclusion and Prospect

BMB‐ME demonstrates potential neurobehavioral effects associated with anxiety, depression, sedation, and muscle relaxation, which may be linked to its phytochemical constituents, including *n*‐hexadecanoic acid, gamma‐sitosterol, and E,Z‐1,3,12‐nonadecatriene. Although the findings are promising, the study is limited by the use of crude extracts and the lack of evaluation of isolated bioactive compounds, thereby restricting definitive mechanistic interpretation. In addition, reliance on in silico docking and ADMET predictions does not fully establish pharmacological efficacy or in vivo bioavailability. The lack of detailed quantitative profiling of individual phytochemicals further limits precise correlation between specific constituents and observed activities. The molecular docking protocol was validated before analysis, thereby supporting the reliability of the predicted ligand–receptor interactions. However, these results should be considered only as supportive computational evidence and cannot confirm direct receptor involvement or establish the underlying mechanism without further experimental validation. Future research should, therefore, focus on the isolation and characterization of active metabolites, along with their quantitative assessment, to better link chemical composition with biological effects. Comprehensive pharmacokinetic and receptor‐level studies, supported by molecular dynamics simulations and long‐term safety evaluations in appropriate animal models, will be essential to clarify CNS penetration, mechanism of action, and therapeutic potential, ultimately facilitating the development of *B. motleyana* as a standardized neuropharmacological agent.

## Author Contributions

Conceptualization, investigation, software, data curation, formal analysis, and writing original draft: Md. Mahmudul Hasan and Md. Ekramul Haque Ekram; investigation, software, data curation, and formal analysis: Kawsar Al Mahmud and Tanvir Hasan; supervision, project administration, writing the original draft, revision, and editing: Md. Tanveer Ahsan. Md. Mahmudul Hasan and Md. Ekramul Haque Ekram contributed equally to this work.

## Funding

No funding was received for this manuscript.

## Ethics Statement

The ethical review board approved this study: ethical review approval number AERB‐FBSCU‐20250615‐(1).

## Conflicts of Interest

The authors declare no conflicts of interest.

## Data Availability

The data that support the findings of this study are available on request from the corresponding author. The data are not publicly available due to privacy or ethical restrictions.
